# Effect of epidermal growth factor ointment on persistent epithelial defects of the cornea

**DOI:** 10.1186/s12886-020-01408-x

**Published:** 2020-04-15

**Authors:** Hyun Sik Moon, Lan Li, Hyeon Jeong Yoon, Yong Sok Ji, Kyung Chul Yoon

**Affiliations:** Department of Ophthalmology and Research Institute of Medical Sciences, Chonnam National University Medical School and Hospital, 42 Jebong-ro, Dong-gu, Gwangju, 61469 South Korea

**Keywords:** Corneal epithelial defects, Epidermal growth factor, Neurotrophic keratitis, Persistent epithelial defects of the cornea

## Abstract

**Background:**

Healthy corneal epithelium acts as a barrier against damage to the deeper structures in the eye. Failure in the mechanisms of corneal epithelization can lead to persistent epithelial defects of the cornea (PEDs) and can compromise its function. Epidermal growth factor (EGF) promotes the proliferation, migration, and differentiation of epithelial cells, endothelial cells, and fibroblasts during wound healing and may be beneficial in treating patients with PEDs. We, therefore, investigated the effect of EGF ointment on patients with PEDs.

**Methods:**

Fifteen patients with PEDs refractory to conventional treatment were treated twice a day with EGF ointment. Patient demographics and comorbidities were noted. The epithelial healing time was determined along with the primary outcome measures in the areas of the epithelial defects, visual acuity, visual analog scale (VAS) scores, and esthesiometer scores 1 month and 2 months after treatment.

**Results:**

Five eyes of herpetic keratitis (33.3%), 3 eyes of dry eye disease (20.0%), 3 eyes of bacterial keratitis (20.0%), 2 eyes of limbal stem cell deficiency (13.3%), 1 eye of diabetic neurotrophic keratitis (6.7%), and 1 eye of filamentary keratitis (6.7%) were associated with PEDs, respectively. Two months following treatment with EGF ointment, there was a reduction in the area of the epithelial defects (5.7 ± 3.9 to 0.1 ± 0.3 mm^2^) as well as a significant improvement in best-corrected visual acuity (0.9 ± 0.8 to 0.6 ± 0.5 LogMAR) and VAS scores (4.5 ± 1.2 to 2.5 ± 0.7) in 12 eyes (80%). Among these cases, the mean epithelial healing time was 5.5 ± 1.8 weeks. Amniotic membrane transplantation was performed on the remaining 3 (20.0%) patients that did not respond to EGF treatment.

**Conclusions:**

EGF ointment could reduce symptoms and promotes corneal epithelialization of refractory PEDs. It may, therefore, be well-tolerated and a potentially beneficial addition in the management of refractory PEDs.

## Background

Persistent epithelial defects of the cornea (PEDs) can result from trauma to the cornea as seen with corneal abrasions or following surgery. In addition, they can develop in patients with dry eye, exposure keratitis, neurotrophic keratitis, corneal ulcers caused by infectious keratitis, and systemic diseases such as diabetes mellitus [1]. PEDs occur when the total thickness of the epithelium is lost and there is no sign of initiation of epithelial resurfacing by the remaining epithelial cells or intact stem cells at the limbus. As a consequence, the corneal epithelium cannot maintain its normal structure. This can be due to a lack of stimulus from growth factors in the tears, neuropeptides released from the trigeminal nerve, and cytokines [2]. The PEDs are characterized by fluorescein staining of the defect area and ground glass-like opacity surrounding the defect. Complications such as secondary infection, scarring, and perforation can occur if the condition is not treated appropriately [3].

The current available noninvasive conventional treatments for PEDs include the administration of lubricating agents, hyperosmotic agents, autologous serum, and umbilical cord serum, as well as the application of therapeutic contact lenses [4–8]. Surgical treatments include debridement, keratectomy, and amniotic membrane transplantation [9–11]. In spite of these many treatment options, some cases of PEDs do not respond to conventional treatment.

Serum treatment is widely used for many ocular surface diseases such as PEDs, and contains several substances that aid regeneration of the keratoconjunctival epithelium, such as epidermal growth factor (EGF), transforming growth factor-β, vitamin A, fibroblast growth factor and insulin-like growth factor-I [4–7]. In particular, EGF is known to stimulate proliferation, migration, and differentiation of epithelial cells, endothelial cells, and fibroblasts during wound healing and [12–14]. EGF often decreased in cases of impaired wound healing such as chronic wounds and diabetic ulcers, and replenishing these factors have been shown to effectively treat such conditions by stimulating angiogenesis and granulation tissue proliferation. Thus, EGF is already used in other clinical fields such as plastic surgery and orthopedics [15, 16]. EGF may, therefore, benefit patients with PEDs recalcitrant to conventional treatment. Considering the abovementioned factors, the present study aimed to evaluate whether EGF is effective in treating patients with PEDs.

## Methods

### Patients

This case-series study included the off-label ophthalmic applications of EGF, and was conducted in accordance with the ethical guidelines of the Declaration of Helsinki and with approval from the Institutional Review Board at the Chonnam National University Hospital (IRB approval number: CNUH-2019-094). Written informed consent was obtained from each patient before enrollment.

PEDs were defined as corneal defects with a minimum area of 2 mm^2^ that persisted without improvement for more than 2 weeks despite conventional treatment, such as lubricating agents, antibiotic eye drops, and therapeutic contact lenses [2]. Medical records of 15 patients (15 eyes) with PEDs unresponsive to conventional treatment and subsequently treated with topical EGF ointment were retrospectively analyzed. Medical records of individuals who underwent invasive procedures or surgery for corneal diseases failed to visit the outpatient department for greater than 2 months, or discontinued EGF treatment due to side effects like pain or conjunctival hyperemia were excluded from the study.

### Treatment agents

After the diagnosis of PEDs, all patients were instructed to instill preservative-free sodium hyaluronate 0.15% (Hyaluni eye drops 0.15%®, Taejoon Pharmaceutical Co., Ltd., Seoul, Korea) ≥6 times a day, levofloxacin 0.5% (Cravit®, Santen, Tokyo, Japan) 2–4 times a day, and lubricant ointment (Duratears®, Alcon Laboratories, Inc., Fort Worth, TX, USA) nightly as conventional therapy. Loteprednol 5% (Lotemax®, Bausch & Lomb, New York, NY, USA) was added when there was inflammation of the ocular surface. In addition, recombinant human EGF ointment (1 μg/g; Easyef® ointment, Daewoong Pharmaceutical Co., Ltd., Seoul, Korea) was applied to the lower conjunctival fornix twice a day in the eyes of patients with refractory PEDs undergoing conventional therapy.

### Evaluation

Patient characteristics (age, sex, and medical history) and causative diseases of PEDs were investigated. The best-corrected visual acuity (BCVA), visual analog scale (VAS) score, corneal sensation, corneal fluorescein staining, and anterior segment photography were performed at 2-week intervals before and after EGF treatment. All examinations were performed by the same investigators.

Corneal sensation was measured using an esthesiometer (Cochet-Bonnet esthesiometer®, Luneau Ophthalmology, Chartres Cedex, France). The tip of the fully extended nylon filament was applied perpendicular to the surface of the central cornea and advanced steadily. When the subject perceived its presence or exhibited a blink reflex, the length of the filament was recorded (mm). To evaluate the area of the corneal epithelial defects, anterior segment photography of the cornea was performed after administration of 2 μl of 1% fluorescein dye at a magnification of × 10 using a camera attached to a slit-lamp microscope. After measuring the longest linear and vertical diameters (mm) of the epithelial defects, the area of the equivalent rectangle was calculated by multiplying the 2 measured dimensions. Treatment was considered completely healing if all the epithelial defects were healed, and partially healing if the defects decreased in size compared with the baseline, even if the epithelial defect was not completely healed within the study period.

### Statistical analysis

Statistical analysis was performed using SPSS Statistics for Windows, version 18.0 (IBM Corp., Armonk, NY, USA). Data are presented as mean ± standard deviation. The Wilcoxon signed-rank test was used to compare changes in variables before and after treatment. A *p*-value < 0.05 was considered statistically significant.

## Results

The mean age of this study group was 65.9 ± 15.5 years (range, 27–86 years); 9 (60.0%) patients were men, and 6 (40.0%) were women. Six (40.0%) patients had hypertension, and 5 (33.3%) patients had diabetes mellitus. All 15 patients exhibited monocular involvement with 8 (53.3%) patients having lesions in the right eye and 7 (46.7%) patients having lesions in the left eye. The causative conditions of the PEDs varied from herpetic keratitis in 5 eyes (33.3%), dry eye disease in 3 eyes (20.0%), bacterial keratitis in 3 eyes (20.0%), limbal stem cell deficiency in 2 eyes (13.3%), diabetic neurotrophic keratitis in 1 eye (6.7%), and filament keratitis in 1 eye (6.7%). The BCVA (logarithm of the minimum angle of resolution [LogMAR]) was 0.7 ± 0.8 LogMAR, VAS score was 5.0 ± 1.3, corneal sensation was 8.0 ± 3.5 mm, and the area of the epithelial defects was 4.7 ± 2.8 mm^2^ at the time of diagnosis of PEDs. The mean duration from diagnosis of PEDs to initiation of treatment with EGF ointment was 183.5 ± 155.9 days (Table 1).

**Table 1:** Demographics of the patients with persistent epithelial defects of the cornea

Variables	Value
Age, years	65.9 ± 15.5
Sex (Male / Female), n (%)	9 / 6 (60.0 / 40.0)
Laterality (right / left), n (%)	8 / 7 (53.3 / 46.7)
Systemic condition	
Hypertension, n (%)	6 (40.0)
Diabetes mellitus, n (%)	5 (33.3)
Ocular past history	
Herpes keratitis, n (%)	5 (33.3)
Dry eye disease, n (%)	3 (20.0)
Bacterial keratitis, n (%)	3 (20.0)
Limbal stem cell deficiency, n (%)	2 (13.3)
Diabetic neurotrophic keratitis, n (%)	1 (6.7)
Filament keratitis, n (%)	1 (6.7)
Visual acuity at first visit, LogMAR	0.7 ± 0.8
Visual analogue scale score	5.0 ± 1.3
Esthesiometer score, mm	8.0 ± 3.5
Area of corneal epithelial defects, mm^2^	4.7 ± 2.8
Intervals between first visit and EGF ointment use, days	183.5 ± 155.9

*LogMAR* Logarithm of the minimum angle of resolution, *EGF* Epidermal growth factor

Corneal epithelial defects were completely healed in 11 eyes (73.3%). Among these individuals, the edema of the corneal epithelium and stroma improved within 2 months after treatment and the mean duration of complete epithelial healing was 5.5 ± 1.8 weeks. One eye (6.7%) had partial improvement with a decrease in corneal epithelial defect size and partial lowering in corneal edema.

**Fig. 1:**
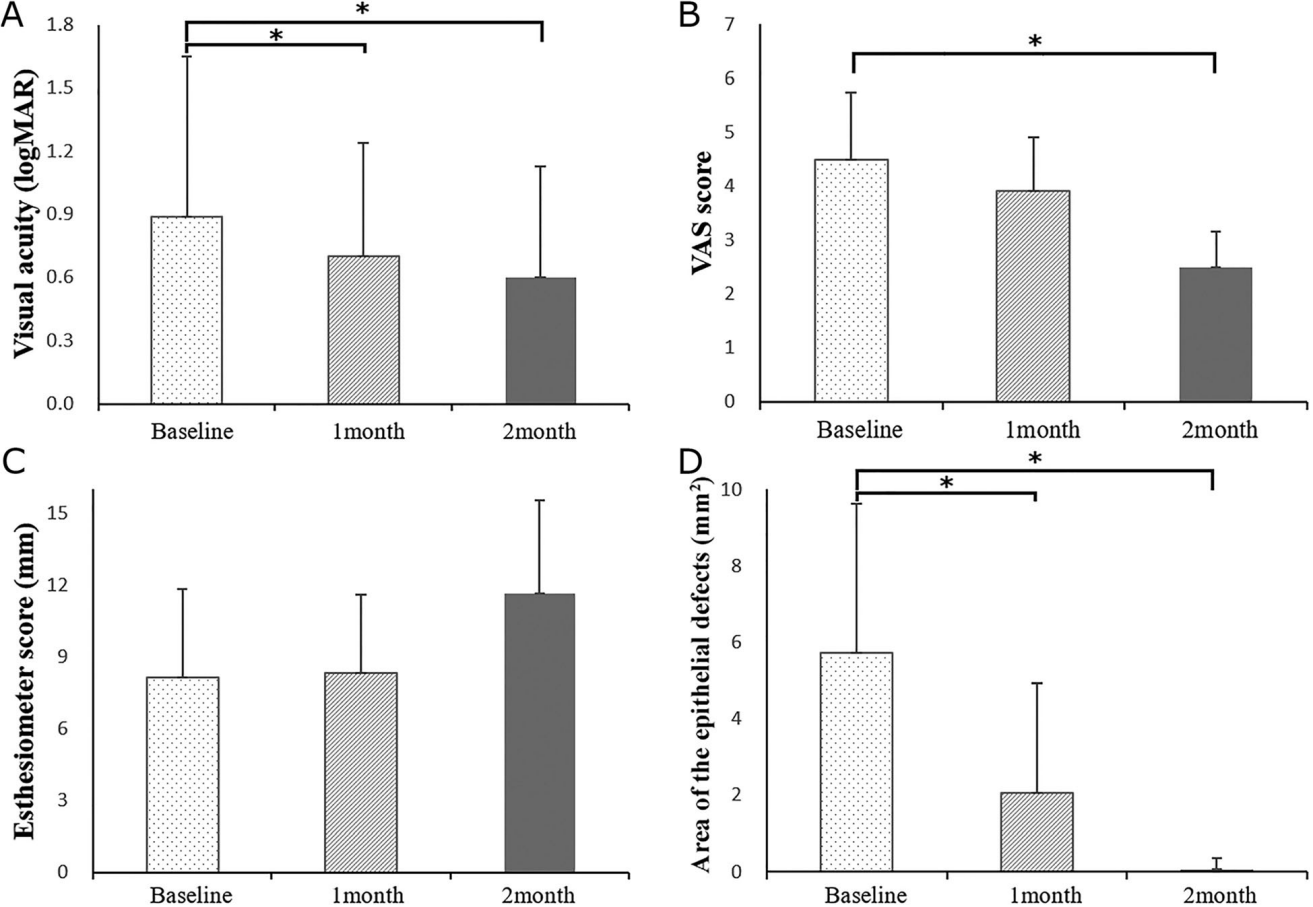
Visual acuity, visual analogue scale (VAS) score, corneal sensation and the area of the epithelial defects before and after 1 and 2 months treated for persistent epithelial defects of the cornea with epidermal growth factor ointment. **a** Visual acuity (LogMAR). **b** VAS score. **c** Esthesiometer score (mm). **d** Area of the epithelial defect (mm^2^). * = significantly different between groups (*p* < 0.05, Wilcoxson-signed ranks test) LogMAR = logarithm of the minimum angle of resolution

At the initiation of treatment with EGF ointment, in the 12 eyes (80.0%) that showed improvement or were completely healed, the BCVA was 0.9 ± 0.8 LogMAR, VAS score was 4.5 ± 1.2, corneal sensation was 8.1 ± 3.7 mm, and the area of the epithelial defects was 5.7 ± 3.9 mm^2^. At 1 and 2 months after treatment with EGF ointment, the BCVA improved to 0.7 ± 0.5 LogMAR (*p* = 0.03) and 0.6 ± 0.5 LogMAR (*p* < 0.01), VAS score decreased to 3.9 ± 1.0 (*p* = 0.07) and 2.5 ± 0.7 (*p* < 0.01), corneal sensation improved to 8.3 ± 3.3 mm (*p* = 0.32) and 11.7 ± 3.9 mm (*p* = 0.18); and area of the epithelial defects decreased to 2.1 ± 2.9 mm^2^ (*p* < 0.01) and 0.1 ± 0.3 mm^2^ (*p* < 0.01, Fig. 1), respectively. The remaining 3 eyes (20.0%) showed no response to EGF treatment, including no improvement in BCVA, VAS score, and no decrease in the area of the corneal epithelial defect; therefore, amniotic membrane transplantation was performed for symptom control (Table 2).

**Table 2:** Clinical characteristics of the patients treated for PEDs with EGF ointment

Patient No.	Primary diseases	Age, years	Sex	Visual acuity, LogMAR		VAS score		Esthesiometer score, mm		Area of epithelial defect, mm^2^		Time of healing, weeks	Outcomes
				Baseline	2 month	Baseline	2 month	Baseline	2 month	Baseline	2 month		
1	Dry eye disease	80 ~ 89	F	1.3	0.6	4	2	15	15	10	0	8	Healed
2	Herpetic keratitis	70 ~ 79	M	1.3	1.2	5	3	15	15	9	0	6	Healed
3	Herpetic keratitis	70 ~ 79	M	0.1	0.1	3	2	5	15	3	0	6	Healed
4	Dry eye disease	50 ~ 59	F	0.1	0.1	2	2	5	15	2	0	6	Healed
5	Herpetic keratitis	70 ~ 79	M	1.3	1.2	6	2	5	15	4	0	8	Healed
6	Dry eye disease	60 ~ 69	F	0.5	0.1	4	2	10	10	3	0	4	Healed
7	Herpetic keratitis	60 ~ 69	M	1.3	1.2	4	2	5	15	3	0	2	Healed
8	Limbal stem cell deficiency	80 ~ 89	F	1.3	1.2	6	3	5	10	2	0	6	Healed
9	Herpetic keratitis	70 ~ 79	F	1.0	1.0	4	5	10	5	4	5	–	AMT
10	Bacterial keratitis	50 ~ 59	M	1.3	1.2	4	3	10	10	10	0	8	Healed
11	Bacterial keratitis	70 ~ 79	M	0.4	0.1	5	3	10	10	4	0	2	Healed
12	Bacterial keratitis	70 ~ 79	F	1.3	0.1	5	2	5	5	4	1	–	Partially healed
13	Diabetic neurotrophic keratitis	60 ~ 69	M	1.7	1.3	6	6	5	5	9	7	–	AMT
14	Limbal stem cell deficiency	20 ~ 29	M	0.2	0.1	6	4	5	5	4	0	4	Healed
15	Filamentary keratitis	40 ~ 49	M	1.7	1.3	6	7	10	10	15	15	–	AMT

*PEDs* Persistent epithelial defects of the cornea, *EGF* Epidermal growth factor, *LogMAR* Logarithm of the minimum angle of resolution, *VAS* Visual analogue scale, *AMT* Amniotic membrane transplantation

A patient with PEDs treated with EGF ointment whose corneal epithelial defect completely healed and who showed improvement in BCVA and VAS scores is described below.

### Case (patient no. 2)

**Fig. 2:**
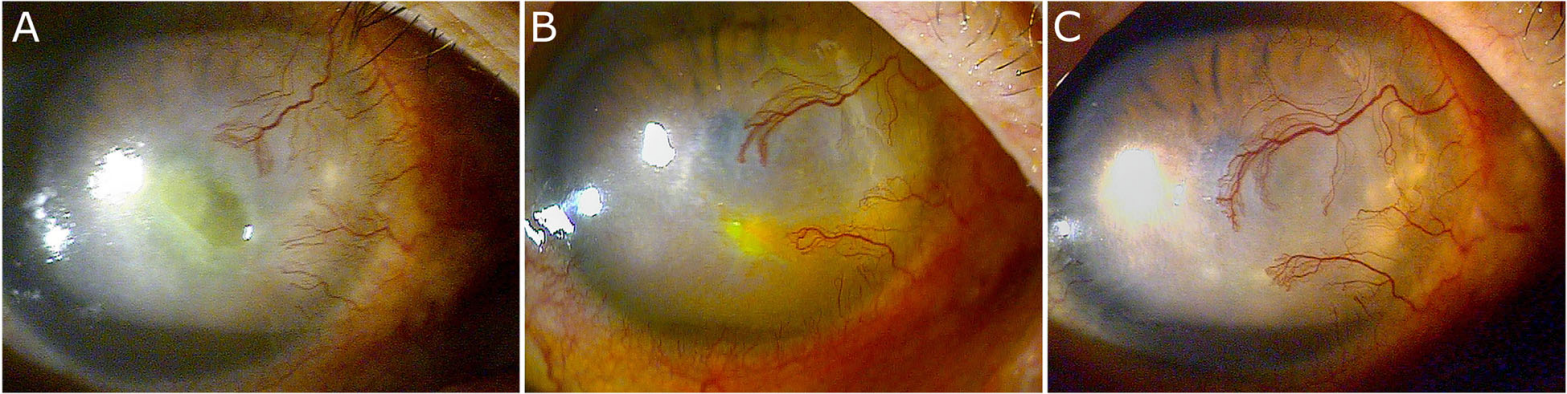
Photographs of slit-lamp examination of persistent epithelial defects of the cornea. **a** Before epidermal growth factor (EGF) therapy, the epithelial defect stained with fluorescein was seen in the para-central cornea with neovascularization of the cornea from the limbus. **b** 4 weeks after EGF therapy, the epithelial defect decreased. **c** 6 weeks after EGF therapy, the epithelial defect was healed with stromal haziness and neovascularization

This case involved a man in his seventies with a long-standing history of dry eye and herpetic keratitis. He exhibited PEDs with peripheral corneal vascularization and scarring in the left eye as a result of his diseases. At the time of diagnosis, his visual acuity was 1.0 LogMAR, and corneal sensation was under 15 mm. He presented with eye pain, photophobia, and epiphora. He had previously undergone treatment with topical levofloxacin 0.5%, topical sodium hyaluronate 0.15%, and lubricant ointment. Despite the treatment, the area of the epithelial defects was 3 × 3 mm^2^, and perilesional corneal edema persisted near the center for at least 4 weeks without any improvement. Therefore, the patient was also instructed to apply EGF ointment twice a day. There was no adverse drug reaction. After 4 weeks of treatment, the lesion improved, and after 6 weeks, the corneal epithelial defects were completely healed, with stromal haze and peripheral corneal vascularization (Fig. 2).

## Discussion

In the present study, we investigated the effect of EGF ointment in patients with PEDs unresponsive to conventional treatment. The EGF ointment was applied twice a day in conjunction with conventional treatment. Two months after treatment with EGF ointment, 12 out of 15 eyes showed significant reductions in epithelial defect size as well as improvements in BCVA and VAS scores.

EGF, a 53 amino acid-containing protein, can affect corneal epithelial healing. It has been demonstrated to be a powerful mitogen of epidermal, epithelial, and endothelial cells in vivo as well as in vitro studies. Furthermore, it was shown that EGF plays a role in physiological and pathological processes, such as embryogenesis, growth, remodeling, and regeneration of tissues, and neoplasm formation [12]. Recombinant human EGF has been produced using human EGF via genetic engineering. Briefly, a microorganism is injected with the human EGF gene to stimulate EGF protein production. Following this, the pure protein is separated and purified. Therefore, mass production in this way is now possible [17, 18].

Several studies have shown the importance of EGF in corneal epithelial healing. Nakamura et al. [19] first identified the role of EGF in the treatment of epithelial defects by demonstrating that inhibitors of EGF receptors prevented the healing of the epithelial defects in vivo. This study has shown that increased EGF levels in the cornea play an essential role in the proliferation and differentiation of cells involved in wound healing after corneal epithelial injury, in addition to maintaining the structural integrity of normal corneal thickness. In another case report, a 79-year-old patient with lung cancer was administered erlotinib for 3 months and PEDs developed; however, the lesion improved 2 weeks after discontinuation of treatment [20]. In the other case report, a 62-year-old patient with colorectal cancer exhibiting multi-organ metastases was administered EGF eye drops (0.05 mg/ml, 8/day) to treat binocular refractory corneal epithelial defects during cetuximab treatment. Epithelial defects were completely healed in the left and right eyes after 7 and 19 days of therapy, respectively [21].

In addition to these, a study using rabbit corneal cells showed that EGF, at certain concentrations, promoted the regeneration of the corneal epithelial and endothelial cells [14]. In another study involving rabbits with recurrent corneal erosions, which were treated with mouse-derived EGF eye drops (0.05 mg/ml, 4/day), the corneal epithelium was found to be regenerated on histological examination; however, a relapse occurred when the treatment was stopped due to weak attachment of the cornea to the basement membrane of the injured stroma. These findings suggest that therapy with EGF alone might be insufficient [22]. Another study using a rabbit model of corneal epithelium defects that was treated with gelatin hydrogel contact lenses, which continuously secrete EGF, reported that these contact lenses had a faster therapeutic effect than EGF eye drops [23]. Further, effective treatment using EGF eye drops (0.05 mg/ml, 3–6/day) for patients with PEDs refractory to conventional therapy [24], and effective fibrovascularization of porous polyethylene orbital implants with Easyef® ointment treatment have been reported [25].

In this study, we used EGF agent in the form of ointment. EGF ointment has been shown to be effective for wound healing without systemic side-effects in multiple clinical trials of dermatologic field [26, 27]. In addition, the formulation of ointment is commercially available, which reduces the inconvenience of drug preparation in the form of eye drop. Patients showed significant improvements in BCVA as well as had reduced corneal epithelial defect size at 1 month after treatment. At 2 months following treatment, these changes, as well as improvements in VAS scores, were seen in 80% of the patients. The patients, who did not respond to conventional treatment for a long period of time, 183.5 days, showed the regression of PEDs within 1 month, suggesting EGF treatment would be effective for regression of PEDs in these cases. Moreover, these results agree with those of previous studies where patients were administered EGF with a higher frequency [21, 24].

In previous studies, the ophthalmic application of EGF was mainly limited to evaluation of the recovery of corneal epithelial defects. In this preliminary case-series study, EGF was applied not only to the treatment of epithelial resurfacing but also to the treatment of other corneal surface diseases such as neurotrophic condition and limbal stem cell deficiency, which are other types of corneal epithelial defects. Additionally, our study had a long follow-up time, so possible lag time in treatment could be overcome. Furthermore, factors that had more practical implications such as vision and eye pain were also evaluated, unlike in other studies. We believe that EGF ointment could be a therapeutic option for the management of refractory PEDs.

There are several limitations to this study. The first limitation is that the study design was a case series and lacked a comparison group. The study, therefore, had low internal validity. The second limitation of the study was the small sample size and thus this patient population may not be truly representative of patients with PED. Additionally, the observation might be a statistical artifact generated from the small sample size. Future analytic studies should be conducted by comparing the intervention group with a control group and employing large sample sizes and various causative conditions for PEDs to properly determine the treatment effect.

## Conclusions

EGF ointment promoted corneal epithelial healing, improved visual acuity, and mitigated ocular symptoms in patients with PEDs refractory to conventional therapy. These findings indicate that EGF ointment is potentially beneficial in the management of refractory PEDs.

## Data Availability

The datasets used and/or analyzed during the current study are available from the corresponding author on reasonable request.

## Abbreviations

BCVA: Best-corrected visual acuity
EGF: Epidermal growth factor
LogMAR: Logarithm of the minimum angle of resolution
PEDs: Persistent epithelial defects of the cornea
VAS: Visual analog scale
